# Supplementary material for: The microbiome biomarkers of pregnant women’s vaginal area predict preterm prelabor rupture in Western China
Source: Front Cell Infect Microbiol. 2024 Oct 31;14:1471027. doi: 10.3389/fcimb.2024.1471027 (PMC11560878; doi:10.3389/fcimb.2024.1471027)
Supplement: Supplementary file 1 [file DataSheet1.zip › compare_1/Community/Pieplot/order/sample/C17.order.pieplot.pdf]

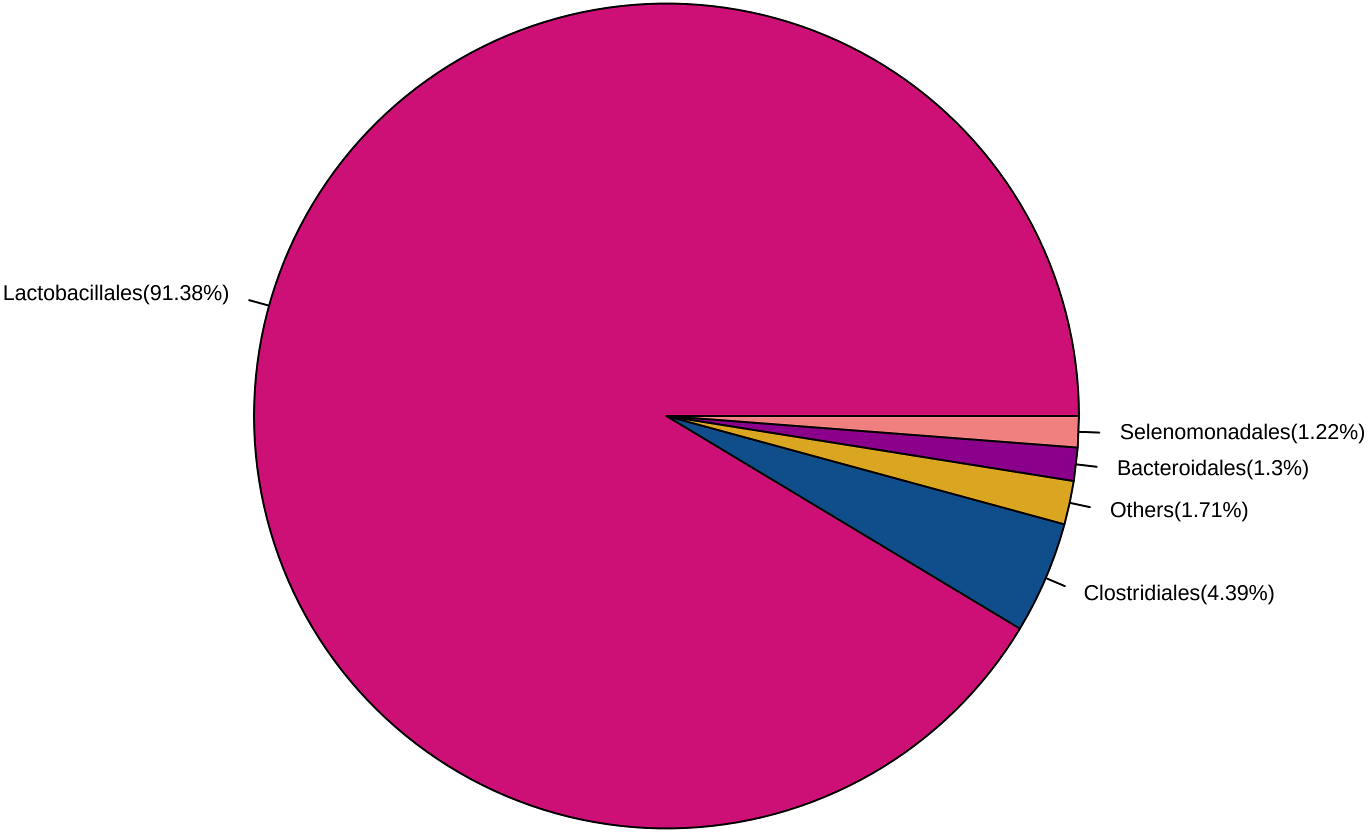

Lactobacillales(91.38%)
Selenomonadales(1.22%)
Bacteroidales(1.3%)
Others(1.71%)
Clostridiales(4.39%)
